# Get BusActive!: Protocol of a single-blinded randomised controlled trial incentivising public transport use for physical activity gain among young people and adults

**DOI:** 10.1016/j.conctc.2024.101367

**Published:** 2024-09-11

**Authors:** Melanie J. Sharman, Oliver Stanesby, Kim A. Jose, Stephen Greaves, Anna Timperio, Elizabeth Reid, Lisa Stafford, Petr Otahal, Verity J. Cleland

**Affiliations:** a Menzies Institute for Medical Research, University of Tasmania, Hobart, Tasmania, Australia; b Institute of Transport and Logistic Studies, The University of Sydney, Sydney, New South Wales, Australia; c School of Exercise & Nutrition Science, Deakin University, Melbourne, Victoria, Australia; d School of Geography, Planning and Spatial Sciences, University of Tasmania, Hobart, Tasmania, Australia

**Keywords:** Transportation facilities, Behaviour and behaviour mechanisms, Economics, Exercise, Sedentary behaviour, Walking

## Abstract

**Background:**

Population level physical activity generally does not meet recommended targets. Compared with private motor vehicle users, public transport users tend to be more physically active and financial incentives may encourage more public transport use, but these relationships are under-investigated. This paper describes the protocol of a randomised controlled trial that aimed to determine the effect of financially incentivising public transport use on physical activity in a regional Australian setting.

**Methods:**

*Get BusActive!* is a 9.5-month single-blinded randomised controlled trial. A convenience sample of Tasmanians aged ≥15 years will be randomised to a 14-week incentive-based intervention (bus trip target attainment rewarded by bus trip credits and weekly supportive text messages) or an active control following baseline measures and will be followed up ∼24 weeks later (maintenance phase). Both groups will receive written physical activity guidelines. The primary outcome is change in accelerometer-measured steps/day from baseline to immediately post intervention phase and maintenance phase. Secondary outcomes are change in: smartcard-measured bus trips/week; measured and self-reported minutes/week of physical activity and sitting; transport-related behaviour (using one-week travel diary), perspectives (e.g. enablers/barriers) and costs; health. Linear mixed model regression will determine group differences. Participant-level process evaluation will be conducted and intervention cost to the public transport provider determined.

**Conclusion:**

*Get BusActive!* will fill an important knowledge gap about the causal relationship between financially incentivised public transport use and physical activity—the findings will benefit health and transport-related decision makers.

**Trial registration:**

ACTRN12623000613606.

**Universal trial number:**

U1111-1292-3414.

## Highlights

• Most young people and adults are insufficiently physically active for health.
• Public transport users tend to be more physically active than motor vehicle users.
• Financially incentivising public transport use increases patronage.
• The link between incentivised public transport and physical activity is unknown.
• GetBusActive!—a world first randomised controlled trial will fill this evidence gap.

## 1 Background

Continuation of current trends in physical inactivity is projected to result in 500 million new cases of non-communicable diseases and INT$520 billion in direct healthcare costs by 2030 [1]. Recent estimates show 81 % of 11–17 years and 27.5 % of adults globally do not meet physical activity targets [2,3]. Although non-communicable disease prevalence attributable to physical inactivity is estimated to be highest in low-middle income countries, the cost burden is proportionately greater in higher income countries [1].

Transport-related physical activity is receiving more attention in part because targeting leisure-time physical activity over the decades has not made measurable differences to population-level physical activity [4]. Fifteen minutes of daily physical activity reduces all-cause mortality risk by 14 %, cardiovascular disease risk by 19 %, ischemic heart disease risk by 25 % and increases life expectancy by three years, so short walks to and/or from public transport stops can be life-saving [5]. Transport-related physical activity also offers an important environmental co-benefits if it leads to less private motor vehicle use [6]. While transport-related physical activity is often discretionary, travelling from place to place (e.g. to/from work, school, services, shops, social/recreational activity) is a necessary behaviour for most. Dual process theory purports that individuals are likely to sustain physical activity if attached to a habitual pattern (i.e., travel) [7]. Yet evidence of effective and scalable strategies for increasing transport-related physical activity is sparse.

A 2012 review of nine studies (all observational conducted in the USA, UK and one in Australia) on physical activity associated with public transport use, showed that public transport users could accumulate an additional 33 min of daily walking [8]. Thus, using public transport can make an important contribution to weekly physical activity targets. In 2016 (census day), only 11.5 % of Australian commuters (employed people 15^+^ years) used public transport to get to work, which dropped to 4.6 % in 2021 likely due to the COVID-19 pandemic [9]. Consequently, there is plenty of scope to improve population level physical activity through public transport use. However, knowledge gaps remain regarding the causal relationship between public transport and physical activity and which strategies best increase transport-related physical activity.

Behavioural economics theory purports that price influences behaviour and choice [10,11]. There is some evidence that financial incentives (e.g. fare reduction) may positively influence behaviours such as physical activity and public transport use [[12], [13], [14], [15]]. There is also evidence of community support for financial incentives to increase public transport use, with a cross-sectional survey finding that financial incentives was rated third of 10 strategies most likely to increase public transport use in an Australian regional setting [16]. In the same setting, young people and people with a disability said that the cost of public transport was a barrier [17]. Taken together, financially incentivising public transport use and physical activity is a promising strategy for improving health, but randomised controlled trials are needed to establish effectiveness.

Responding to the evidence gap and an identified partner (a public transport provider, local and state government) need, in 2019 *trips4health* (an Australian based study) was the first randomised controlled trial to investigate the causal link between financially incentivised public transport and physical activity. However, *trips4health* was abandoned in mid-2020 due to substantial social and behavioural impacts of the COVID-19 pandemic. At trial cessation, one third (n = 110) of the target sample had completed a 16-week intervention (or equivalent control) phase, therefore providing only preliminary and process evaluation findings [18]. Financial incentives appeared to increase public transport use and transport-related physical activity, but the impact on total physical activity was less clear [19,20]. Although *trips4health* demonstrated strong fidelity and feasibility, process evaluation data highlighted that some population groups were under-represented (e.g. men, and those less educated) and some aspects of the intervention could be improved [18].

*Get BusActive!* builds on preliminary and process evaluation findings from *trips4health* and partner feedback and has refined inclusion criteria, recruitment approaches, outcome measures and data collection methods. Co-design processes with consumers/community and end-users are embedded to support study participation and subsequent scale-up. *GetBusActive!* aims to determine the causal relationship between financially incentivised public transport and physical activity and its longer-term impacts in a regional setting.

## 2 Objectives/Hypothesis

The primary hypothesis is: A public transport incentives scheme will increase daily steps (accelerometer-measured) compared to a control group. Secondary hypotheses are: A public transport incentives scheme will increase public transport use (objectively measured) and transport-related physical activity (self-reported) compared to a control group.

## 3 Trial design

Single-blinded parallel group randomised controlled trial.

## 4 Materials and methods

### 4.1 Study setting

**Fig. 1:**
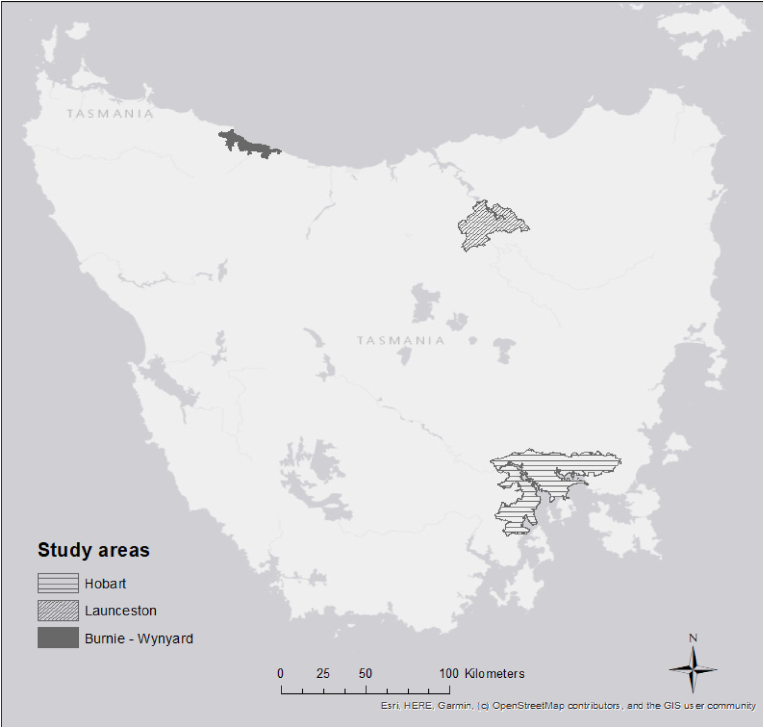
Study setting.

The study will occur in Tasmania, Australia. Tasmania is an island state with a population of approximately 555,000 (460,344 ≥ 15 years). Public transport is provided predominantly by bus, mostly by Metro Tasmania, with services delivered in three of the main population centres—Greater Hobart (population: 226,653), Launceston (population: 90,953) and Burnie (population: 19,919) [21] (Fig. 1). Tasmania has one of the lowest rates of public transport use in Australia with only 3 % and 3.2 % of employed people 15 years^+^ using public transport to get to work in 2016 and 2021 respectively [9]. Unequal and inequitable access to Tasmanian public transport and limitations in service provision contribute to low patronage [17,22,23]. Further, only 26 % of Tasmanians (15^+^ years) self-reported meeting weekly physical activity targets in 2021 [24].

### 4.2 Eligibility criteria

Potential participants will be screened by online survey or telephone. The self-assessed inclusion and exclusion criteria are listed in Box 1. Those eligible will receive an information sheet and provide online or verbal consent.

Box 1 **Self-assessed inclusion and exclusion criteria**

**Table:** 

Inclusion criteria
• ≥ 15 years
• Tasmanian resident
• sufficient English proficiency to provide informed consent
• possession of a mobile phone and active email address
• ownership of or willingness to obtain a Metro Greencard (travel smartcard)
• willingness to give permission for the researchers to access smartcard data
• currently making no more than five Metro bus trips/week
• able to reasonably access a Metro bus service
• currently making trips by motor vehicle (including motorcycle) that could be made by bus
Exclusion criteria
• intending to move house or work location within the study period whereby a Metro bus service will be inaccessible
• currently engaged in or planning to engage in other public transport incentives program
• pregnancy
• a health condition that prevents walking
• a health condition that prevents bus use
• a planned activity that would prevent bus use for greater than four weeks during the 14-week intervention phase of the 38-week study period (e.g. surgery, extended holiday)

Alt-text: Box 1

### 4.3 Interventions

The intervention group will receive a 14-week intervention whereby they are financially incentivised with smartcard credit to meet weekly bus trip targets, supplemented by weekly supportive text messages (Table 1). Intervention participants will also receive Australia's physical activity and sedentary behaviour guidelines for adults (≥18 years) or for young people (<18 years), as relevant [25,26]. *Get BusActive!* is based on the *trips4health* protocol but with enhancements informed by preliminary and process evaluation findings [[18], [19], [20],^27^]. Using a gain-framed approach (rewarding positive behaviours), targets increase progressively, with corresponding incentive increases. *trips4health* data indicated a levelling out in target attainment beyond three trips/week, so a maximum trip increase target of three/week is scheduled, with a stretch goal of four/week [19]. While there is no consensus on minimum time for habit formation, at least 66 days (9.4 weeks) are likely needed [28]. Subsequently, a 14-week intervention phase has been chosen which allows 10 weeks for habit formation and four weeks for maintenance (‘tapering off’), where contact and support is gradually withdrawn in preparation for intervention cessation [28].

**Table 1:** *Get BusActive!* intervention schedule.

	Intervention phase	Bus trip target increase p/wk	Incentive – bus trip credits	$ value (all zones Child/student^a^)	$ value (all zones Adult)	$ value (all zones Adult concession)	Text message frequency
Week 1	Getting started	1	1	1.52	5.76	1.92	x 2
Week 2	Getting started	1	1	1.52	5.76	1.92	x 2
Week 3	Getting started	1	1	1.52	5.76	1.92	x 2
Week 4	Building up	2	2	3.04	11.52	3.84	x 2
Week 5	Building up	2	2	3.04	11.52	3.84	x 2
Week 6	Building up	2	2	3.04	11.52	3.84	x 2
Week 7	Moving along	3	3	4.56	17.28	5.76	x 2
Week 8	Moving along	3	3	4.56	17.28	5.76	x 2
Week 9	Moving along	3	3	4.56	17.28	5.76	x 2
Week 10	Aiming high	4	5	7.60	28.8	9.6	x 2
Week 11	Maintenance	3	3	4.56	17.28	5.76	x 2
Week 12	Maintenance	3	3	4.56	17.28	5.76	x 2
Week 13	Tapering	3	2	3.04	11.52	3.84	x 1
Week 14	Tapering	3	1	1.52	5.76	1.92	x 1
***Total***		**34**	**32**	**$48.64** (AUD)	**184.32** (AUD)	**$61.44** (AUD)	**28**

Value of incentive = cost of participant’s usual fare type times number of bus trip credits allocated aeligible for child/student fare up until end of year in which passenger turns 18 years old.

To assist participants in achieving weekly targets and consistent with best practice, incentives will be supported by weekly text messages (two per week for weeks 1–12 and once per week in weeks 13 and 14) that target other behaviour change techniques (e.g. planning, goal setting) [29]. Text messages may help counter the potential risk that incentives alone could undermine intrinsic motivation in the absence of broader behavioural support [30]. In *trips4health*, text messages were well-received, but some participants recommended personalising the messages to increase salience and impact–consequently text messages will be co-designed with end-users and informed by habit formation theory [28,31], the Behaviour Change Technique Taxonomy [32], our previous qualitative work [16,33] and a relevant incentive-based study targeting leisure-time physical activity [12].

Active control participants will receive the same physical activity guidelines as outlined for the intervention group [25,26]. Cognitive strategies such as providing physical activity guidelines are unlikely to influence physical activity behaviour [34]—the guidelines will be provided to help engage and retain participants.

### 4.4 Outcomes

The primary and secondary outcomes and timing of measures are summarised in Table 2. The primary outcome is change in accelerometer-measured average daily step count (steps/day) over seven days. There are 15 secondary outcomes relating to travel behaviour and perspectives, physical activity, sedentary time (sitting), health, transport behaviour economics and intervention implementation costs.

**Table 2:** Primary and secondary outcomes and timing of measures.

Outcome (change in …)	Timing of measure	T1	T1-T2	T2	T3
Primary outcome					
Accelerometer measured steps/day averaged over seven days		X		X	X
Secondary outcomes					
Accelerometer measured minutes/week of physical activity		X		X	X
Accelerometer measured minutes/week of sitting		X		X	X
Smartcard measured bus trips/week			X	X	X
Smartcard measured costs to public transport provider			X		
Self-reported (travel diary) trips/week by mode, frequency and duration		X		X	X
Self-reported (travel diary) commute time/week by mode		X		X	X
Self-reported (survey) perspectives on travel behaviour (e.g. enablers/barriers)		X		X	X
Self-reported (survey) out of pocket transport-related expenses		X		X	X
Self-reported (survey) work productivity		X		X	X
Self-reported (survey) commute time to work/study		X		X	X
Self-reported (survey) commute by mode to work/study		X		X	X
Self-reported (survey) physical activity minutes/week by type (transport, leisure, occupational, domestic, total)		X		X	X
Self-reported (survey) sitting hours/day		X		X	X
Self-reported (survey) body mass index via self-measured height and weight		X		X	X
Self-reported (survey) health		X		X	X

T1/Timepoint 1: Baseline; T2/Timepoint 2: Immediately after completion of the14-week intervention phase (∼week 15); T3/Timepoint 3: Immediately after completion of the 24-week maintenance phase (∼week 39).

### 4.5 Participant timeline

**Fig. 2:**
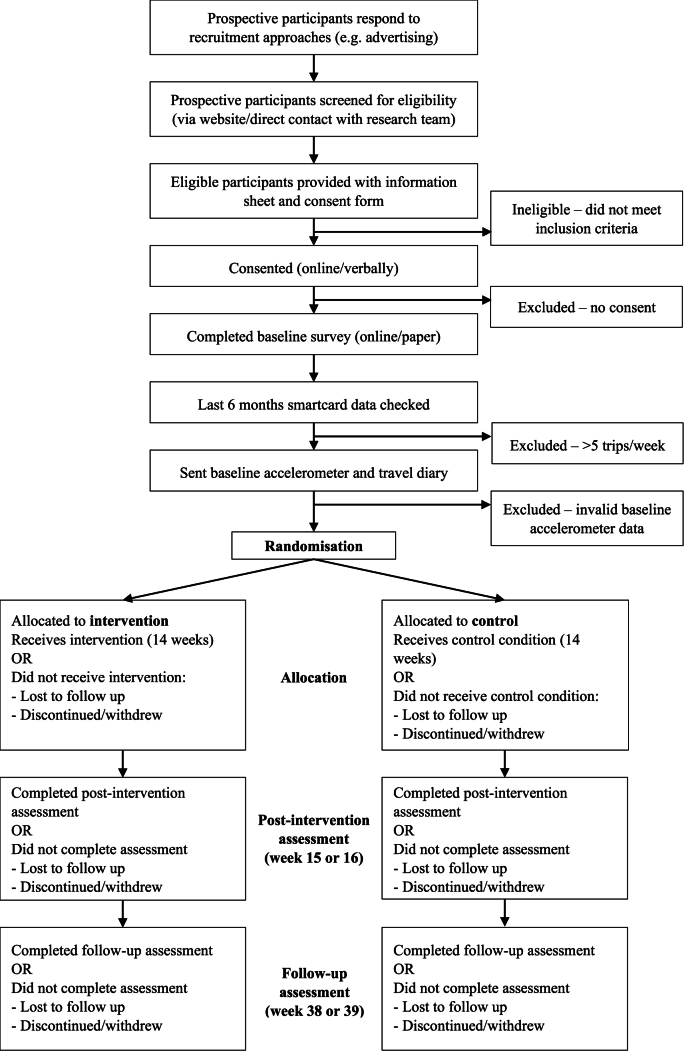
Participant flowchart.

The participant flowchart is summarised in Fig. 2.

### 4.6 Sample size

At least 345 participants will be randomly allocated across both study arms—an oversampling of 15 % to allow for attrition (based conservatively on *trips4health* and the relevant ACHIEVE study [12,18]) leaving a minimum of 300 participants. Three hundred participants will provide 80 % power (two-sided α = 0.05) to detect a 34 % change (∼2500 steps/day) in daily steps in the intervention group relative to controls (primary outcome) and meaningful increases (13–29 %) in secondary outcomes. The power calculation is based on the standard deviation of change in the physical activity outcomes, log-transformed due to the right-skewed nature of physical activity data (Table 3).

**Table 3:** Increase in outcomes detected with n = 300.

Outcomes	Standard deviation of change (log scale)^a^	Relative increase in outcome
Accelerometer (steps/day)	0.336	11.5 %
Transport-related physical activity (hours/week)	0.790	29.1 %
Bus use in week (trips/day)	0.390	13.4 %

a Standard deviation estimates from *trips4health*.

### 4.7 Recruitment

In *trips4health* there was an over-representation of females (69 %) and those university educated (55 %) compared with the Greater Hobart population where the study was located (52 % females, 17 % university educated) [18]. A consumer advisory group will co-design recruitment approaches and materials for *Get BusActive!* to maximise participant reach and diversity. The consumer advisory group (chaired by a lead investigator) will comprise 6–10 diverse members from the study area.

A range of recruitment methods will be used e.g. social and traditional media, the Metro website and Metro bus advertising, cross-promotion via other relevant surveys, Tasmanian Government staff newsletter; University of Tasmania (UTAS) staff intranet; local government (particularly councils in the catchment area); libraries and relevant community-based organisations and advising participants from our previous transport-related studies who had indicated their interest to be informed of future projects. Interested individuals will be directed to a study website or telephone number for screening.

To support recruitment and retention, participants will be compensated (in the form of a gift card) for completing each of the three assessments—T1 = $20 gift card; T2 = $35 gift card; T3 = $45 gift card (total $100). Another retention strategy is allowing pauses in study participation for up to four weeks in total (minimum one week for each pause) during the 14-week intervention period if circumstances preventing bus use arise (e.g. travel, prolonged illness).

### 4.8 Assignment of interventions

Once participants have completed T1 they will be randomly allocated to the control or intervention arm. Randomisation will be allocated on a 1:1 ratio in blocks of four, without stratification. The allocation sequence will be created using a pseudo-random algorithm in Stata (17.0, StataCorp LLC, College Station, TX) with a true random seed number between 1 and 1,000,000 [35]. The allocation sequence will be executed in Research Electronic Data Capture (REDCap, Version 13.7.31, Nashville, Tennessee, USA). Research team members who enrol participants in the respective study arms will not have access to the randomisation details.

Participants cannot be blinded to treatment allocation given the intervention type. All members of the research team will be blinded except for the research assistants. Unblinded staff will enrol participants, assign participants to the control or intervention arms as per the treatment allocation sequence generated, inform participants of the study requirements according to intervention/control group allocation and be the main contact point for participants. Unblinded staff will not analyse data. The research team member who creates the randomisation sequence will not contact participants, nor administer data collection. All other research team members (investigators, data analysts, postgraduate students) will be blinded. The need to unblind other research team members is unlikely.

### 4.9 Data collection, management and analysis

Participants will be asked to complete three assessments (Table 2).

#### 4.9.1 Accelerometer

At T1, T2 and T3, participants will be asked to wear a hip-worn Actigraph wGT3X-BT, GT3X + or GT3X accelerometer for seven consecutive days, as recommended [36,37]. Accelerometers indicate total ambulatory (walking) activity, the physical activity behaviour most likely to be impacted by and most closely aligned to the intervention, via estimation of steps. Accelerometers provide valid and reliable measures of steps, physical activity intensity and duration and sedentary time [[38], [39], [40], [41]]. Minimum wear time criteria to be included in analyses is 8 h/day for ≥ four days with non-wear defined as ≥ 60 min of continuous zero counts [36,37].

Accelerometers will be mailed to participants in padded postage packs. Instructions provided (written and/or verbal) will cover—wearing and returning the accelerometer, research team contact details, diary completion for noting date and time of wear and notable comments (e.g. unwell, forgot to wear) and a video link on correct accelerometer use. Monitors not returned will be followed up via telephone and email until received.

#### 4.9.2 Bus trip data

Permission to use participants' Metro smartcard data is an eligibility requirement—this eligibility criteria was not a major participation barrier in *trips4health* (only 12/444 potential participants were either unwilling to get a smartcard or share their smartcard data). Smartcard data will be retrieved for the six months prior to study commencement (for participants with an existing smartcard) to objectively assess participants’ usual bus use and confirm eligibility.

A second use of smartcard data is to determine bus trip target attainment during the 14-week intervention phase, with Metro Tasmania allocating credit to participants meeting the targets—a feasible and acceptable process to participants and Metro Tasmania established in *trips4health* [18]. Bus trip credit will be commensurate with fare type (e.g. Adult, Adult Concession, Child/student; across zones 1–3). Participants will be notified via a weekly email whether they met their target, (any) credit they will receive and the next target.

A final use of smartcard data will be to determine any bus use change within and between the control and intervention groups.

Smartcard data collected will include: smartcard number; date, time and fare amount charged ($) at each boarding.

#### 4.9.3 Surveys

Surveys will be electronic, with hard copies provided upon request. The surveys will include physical activity (T1, T2, T3), demographic (T1, T2, T3), health (T1, T2, T3), travel (T1, T2, T3), and changing circumstances (T2, T3) questions. The surveys have been adapted for young people as appropriate—removing questions suitable for adults only (e.g. marital status, height/weight, certain health conditions/behaviours) or including questions relevant to young people only (e.g. physical activity within the school setting).

• *Physical activity questions:* The International Physical Activity Questionnaire – Long form (IPAQ-L) is reliable and widely used and will capture transport-related, leisure time, occupational, domestic and total physical activity and sedentary time at T1, T2 and T3 [42]. For young people only, questions related to physical activity within the school setting from the IPAQ-A were included [43].
• *Demographic questions:* At T1 only: gender, date of birth, and language spoken at home. At T1, T2 and T3: highest level of education, student status, employment status, household composition, residential and work/study address, and income. Additionally, adults at T1, T2 and T3 will be asked about marital status and work from home arrangements.
• *Health-related questions:* Adult participants only: height and weight using standard measures; if they have ever (and if current) been told by a doctor that they have high blood pressure, heart disease, stroke, diabetes, cancer, osteoporosis, depression or anxiety, or arthritis (Tasmanian Population Health Survey items [44]); current smoking [44]; illness/injury/disability that limits physical activity or bus use. All participants will be asked to rate their health ranging from excellent to poor. Health-related data will be collected at T1, T2 and T3.
• *Travel-related questions:* At T1, T2 and T3 motorised vehicle access and ownership, access to equipment for active travel e.g. bicycle, driver's license, transport mode and frequency, time and distance to nearest bus stop (from home and work/study) and enablers/barriers of bus use. A simple paper-based seven-day travel diary completed at T1, T2 and T3 will capture mode, duration, and frequency of public, private, and active travel. Use of TravelVu will be encouraged—a freely available travel app that captures travel information per trip which will aid diary completion through better recall [45]. If participants use TravelVu they will be encouraged to send these data as a.csv file by email to a designated staff member.
• *Economic evaluation questions:* Adult participants only: time spent commuting, use of time whilst commuting, work productivity and transport-related costs (e.g. walking equipment, fuel, parking) at T1, T2, T3
• *Changing circumstances questions:* At T2 and T3: changes in health, home, or work circumstances, travel characteristics, or contact details.
• *Process evaluation:* At T1, T2 and T3, participants will be asked to evaluate study materials, correspondence, intervention impact, report on their motivations for participating and staying involved in the study and provide general feedback.

The survey will be pilot tested on a convenience sample of individuals ≥15 years (e.g. family members and colleagues of the research team, staff from partner organisations, Consumer Advisory Group members) as another strategy to ensure suitability, completion and retention. Pilot testing data will not be retained.

### 4.10 Statistical methods

Quantitative analyses will be conducted using Stata (17.0, StataCorp LLC, College Station, TX). Descriptive statistics will characterise participants, acceptability, and outcomes according to group allocation. Primary intention-to-treat analyses will focus on comparing change in daily steps from baseline (T1) to 14 weeks (T2). A random-intercepts linear regression model with covariates for T1 steps, time (1 = T2, 0 = T1), baseline steps x time, and group (1 = intervention, 0 = control) x time will be estimated. The efficacy of intervention (primary outcome) at T2 will be assessed from the coefficient of group × time (equivalent to an analysis of covariance, and to a change scores method when a T1 steps covariate is included, though standard errors may differ slightly). The advantage of this model is that it can be readily extended to analysis of a third time-point to test whether intervention efficacy is maintained at 39 weeks (T3). Prior to analysis, right-skewed physical activity data will be transformed using a logarithmic transformation, which worked well with the *trips4health* trial data. All models will be estimated without and with adjustment for T1 participant characteristics that differ materially between groups.

Individual-level time spent commuting, use of time whilst commuting, work productivity and financial outlay for transport (e.g. walking equipment, fuel, parking) will be analysed as part of the economic evaluation. Non-research related costs and benefits of intervention implementation relative to the control group will be assessed. Modelled analyses will assess the financial implications of broader implementation rollout and upscaling of the intervention.

Process evaluation will assess the fidelity and quality of implementation, clarify causal mechanisms and identify contextual factors associated with outcome variation. The Medical Research Council (United Kingdom) framework will underpin the process evaluation as it is designed to examine complex public health interventions [46]. Process evaluation will involve face-to-face or telephone/video interviews (n = 10) with intervention participants (who did and did not reach bus targets). Administrative and research data may be used in process evaluation analysis. Qualitative process evaluation data will be analysed thematically using NVivo software (QSR International, Doncaster, Victoria, Australia).

### 4.11 Data monitoring

#### 4.11.1 Harms

Physical activity intervention studies pose minimal risk to participants [47]. The most common (but unlikely) physical activity intervention study-related adverse events are minor musculoskeletal injuries [47]. Adverse events will be logged in REDCap, reviewed by the nominated clinician chief investigator to determine the appropriate action and reported to the ethics committee. Participants disclosing existing health conditions/injuries that limits bus use will be encouraged to seek advice from their health practitioner before study participation. Participants in the intervention group will be provided with a ‘Checklist’ with tips for safe bus travel; as control group participants are not being encouraged to catch the bus more, they will not receive this checklist.

At study end, participants will be provided with summary health-related information (e.g. physical activity and for adults only body mass index). Participants will be encouraged to share this summary information with their health care practitioner, especially if values are outside normal limits or physical activity targets are unmet.

#### 4.11.2 Auditing

Audits will be conducted after approximately 10 % and then 50 % of the total sample have completed week two of the intervention phase. At each audit, an unblinded staff member will check study operationalisation (e.g. consent forms signed and stored, randomisation process working, surveys complete, data stored appropriately, correct and timely correspondence to participants, bus trip incentives correctly allocated).

### 4.12 Ethics and dissemination

#### 4.12.1 Protocol amendments

Protocol amendments will be submitted to the Tasmanian Health and Medical Human Research Ethics Committee for approval. Where required, relevant parties will be notified of protocol changes. If any protocol changes directly impact participants (e.g. changed eligibility criteria), participants will be asked to re-consent.

#### 4.12.2 Consent

Participants will provide consent electronically (online) or verbally (by a staff member via telephone) using our RedCap data captures system. Consent forms will be stored securely and separate to study data. Due to the intervention type (financial incentive), the extent of data collection, and study length, young people 15–17 years old will be directed to a consent form to be co-signed by a parent/carer. If a young person is living independently from their parent/carer and unable to gain their consent, they are legally able to give their own. In this case a member of the research team will discuss the study requirements with them.

#### 4.12.3 Confidentiality

Research team members will have access to administrative data (e.g. names, contact details) only when necessary. For data cleaning and analysis, research data will be kept separately from administrative information. Only the unique identifier assigned to each participant will be used for data cleaning and analysis. Only investigators will have access to de-identified data. Study-related reports, publications and presentations will not include identifiable information. *Dissemination policy* An integrated knowledge translation plan will be informed by the Canadian Institutes of Health Research Guide to Knowledge Translation Planning [48]. The plan will include identifying the knowledge-user audience (beyond the research team), strategies (diffusion, dissemination, application), expertise (e.g. graphic design), and adequate resource allocation. Findings will be shared through traditional (e.g. journal publications and conference presentations) and non-traditional (e.g. social media) academic pathways. Authorship of academic output will abide by the National Health and Medical Research Council guidelines [49].

Participants will be provided with summary findings at study completion and offered summary personal information including body mass index (adults only) and a visual representation of their bus use and accelerometer derived physical activity. Participants will be alerted to any publicly available reports produced.

## 5 Discussion

*Get BusActive!* will fill an evidence gap regarding the causal relationship between financially incentivised public transport use and physical activity. Although, *trips4health,* was abandoned due to the COVID-19 pandemic, the study was adequately progressed to provide informative preliminary and process evaluation data [18]. These data have been used to improve the study design of *Get BusActive! Get BusActive!* also includes several strategies to mitigate further potential pandemic impacts (e.g. no face-to-face study requirements).

The comprehensive measures collected through *Get BusActive!* (including objectively measured physical activity and bus use) will provide end-users (e.g. policy makers, public health officers) with valuable information to inform evidence-based decision making. End-users have been engaged since study inception (planning phase) and will continue to provide input across the study's life-cycle (ending with the sharing of findings). End-user engagement across the study's life-cycle increases study relevance, broadens reach and fosters more effective implementation of the findings. Engaging decision makers will also ideally lead to much needed “upstream” policy- or environmental-level interventions more likely to positively impact population-level physical activity [50].

However, this study is not without its limitations. The study setting is regional and therefore the findings may not be generalisable to urban environments or other regional settings where differences exist such as in the built environment, public transport provision and cultural and social norms. Further, despite heightened efforts to recruit harder to reach population groups, it is possible that over-representation of certain demographic characteristics will persist—the internal and external validity of the findings will be checked using census data of Tasmanian public transport users and the whole population.

## 6 Conclusions

Given the high global prevalence of physical inactivity and its contribution to morbidity and premature mortality, novel and scalable interventions are needed to address its escalating individual- and societal-level cost. *Get BusActive!* responds to calls for more randomised controlled trial evidence examining the relationship between physical activity and transport behaviour in a real-world setting.

## Ethics approval and consent to participate

This study was ethically approved by the Tasmanian Health and Medical Human Research Ethics Committee (approval number 27961).

## Consent for publication

Not applicable.

## Availability of data and material

De-identified data from *Get BusActive!* will be made available in a protected archive https://researchdata.edu.au/following requests to the data custodians. Data may be used by researchers outside of the investigator team if ethics processes are adhered to and permission for use is granted by the custodians. Analytic code used to conduct analyses in *Get BusActive!* will not be available in a public archive but may be provided by emailing the corresponding author.

## Funding

This work is supported by a Medical Research Future Fund grant administered through the 10.13039/501100000925National Health and Medical Research Council Australia (MRF2016173) and through in-kind support from Metro Tasmania, the 10.13039/501100022513Tasmanian Department of Health (Public Health Services), Tasmanian Collaboration for Health Improvement and the Local Government Association of Tasmania. Verity Cleland is supported by a 10.13039/501100001030National Heart Foundation of Australia Future Leader Fellowship (2021–2024, ID104892).

## CRediT authorship contribution statement

**Melanie J. Sharman:** Writing – original draft, Project administration. **Oliver Stanesby:** Writing – review & editing, Project administration. **Kim A. Jose:** Writing – review & editing, Project administration, Methodology. **Stephen Greaves:** Writing – review & editing, Methodology. **Anna Timperio:** Writing – review & editing, Methodology. **Elizabeth Reid:** Writing – review & editing, Project administration. **Lisa Stafford:** Writing – review & editing, Methodology. **Petr Otahal:** Writing – review & editing, Methodology. **Verity J. Cleland:** Writing – review & editing, Resources, Methodology, Funding acquisition, Conceptualization.

## Declaration of competing interest

The authors declare the following financial interests/personal relationships which may be considered as potential competing interests:This work is supported by a Medical Research Future Fund grant administered through the National Health and Medical Research Council Australia (MRF2016173) and through in-kind support from Metro Tasmania, the Tasmanian Department of Health (Public Health Services), Tasmanian Collaboration for Health Improvement and the Local Government Association of Tasmania. Verity Cleland is supported by a National Heart Foundation of Australia Future Leader Fellowship (2021–2024, ID104892).

## Data Availability

No data was used for the research described in the article.
